# The Effectiveness of Cognitive Behavioral Therapy on Insomnia Severity Among Menopausal Women: A Scoping Review

**DOI:** 10.3390/life14111405

**Published:** 2024-10-31

**Authors:** Anastasia Ntikoudi, Dimitra Anna Owens, Alketa Spyrou, Eleni Evangelou, Eugenia Vlachou

**Affiliations:** 1 Department of Nursing, University of West Attica, 12243 Athens, Greece; aspirou@gmail.com (A.S.); elevagel@uniwa.gr (E.E.); evlachou@uniwa.gr (E.V.); 2 Medical School, National and Kapodistrian University of Athens, 15772 Athens, Greece; dowens@med.uoa.gr

**Keywords:** cognitive behavioral therapy, menopause, insomnia

## Abstract

This review explores the impact of cognitive behavioral therapy for insomnia (CBT-I) on menopausal women suffering from insomnia. The transition to menopause is often accompanies by sleep disturbances, which significantly affect women’s quality of life. This review applies a scoping approach to evaluate randomized controlled trials (RCTs) focused on CBT-I interventions for insomnia among menopausal women. The included studies examined variations in the number of CBT-I sessions, the duration of interventions, and their delivery methods (face-to-face, online, or telephone-based). The results consistently showed that CBT-I significantly improves sleep quality and reduces insomnia severity in menopausal women. CBT-I was particularly effective compared to other interventions such as sleep restriction therapy and sleep hygiene education. Sleep quality improvements were observed to persist for up to six months after treatment. These findings support the use of CBT-I as a first-line intervention for insomnia in menopausal women, offering a sustainable solution with fewer side effects compared to pharmacological treatments. However, the review also highlights the need for further research on CBT-I’s efficacy in diverse populations, as most studies focused on predominantly white and well-educated women.

## 1. Introduction

Menopause is considered the end of the fertile phase of a woman’s life and has a great emotional, social, and physical impact on women. The symptoms of menopause are due to the decline in hormone levels in relation to the menstrual cycle on the circulation. The symptoms that occur during the transition to menopause are divided into three broad categories, which include vasomotor, atrophic, and psychological or sexual symptoms. The vasomotor symptoms include the most common and intense symptoms of perimenopausal and early postmenopausal women, namely hot flushes and night sweats. These symptoms may be accompanied by discomfort, fatigue, and insomnia [1]. At the onset of menopause, the two biggest changes in a woman’s life are a decrease in sleep quality and a decrease in estrogen levels. The latter is likely to bring about symptoms such as vasomotor symptoms, depression, and an increase in anxiety. Sleep quality changes in all menopausal women.

There is an increase in sleep problems at about 30% in pre-menopausal women, reaching a rate of 50% in postmenopausal women. The factors that cause changes in sleep quality vary. In more detail, decreased estradiol levels and increased FSH levels cause disturbances in the secretion of melatonin and circadian hormones, which are responsible for people’s sleep quality. During sleep, sweating and repeated hot flushes are observed, which disturb it, and in fact, very early awakening and the inability to sleep afterwards are sometimes observed [2].

These sleep disturbances in menopausal women lead to poor-quality sleep and consequently to feelings of depression and anxiety. Several mental health problems, in general, can occur during menopause, which is due to the onset of related problems. There is also a link between these categories of problems. For example, anxiety, which is a psychological symptom, and sleep, which is vasomotor, are linked as previously mentioned [3].

A significant indicator of the menopausal transition is sleep disturbance. Various biological and chronobiological elements contribute to this phenomenon, including age-related physiological alterations, menopausal symptoms such as vasomotor symptoms (VMS), negative health perceptions, mood-related symptoms, and co-existing chronic health issues like back pain, musculoskeletal disorders, and osteoarthritis. Additionally, socioeconomic, psychosocial, and racial or ethnic factors also exert an influence [4,5,6,7]. Nonetheless, an independent correlation exists between menopause and sleep disturbances, which is separate from the influence of aging and various confounding variables. In comparison to the prevalence rates of obstructive sleep apnea (16–20%) and restless legs syndrome (20–24%), insomnia emerges as the most prevalent sleep disorder associated with menopause [2,8,9,10,11,12]. In a multiethnic survey (Study of Women’s Health Nationwide [SWAN]) that followed menopausal women for 10 years, 46–48% of menopausal women experienced insomnia, compared with 38% of premenopausal women [13,14].

Insomnia is a clinical condition characterized by difficulty falling asleep, difficulty remaining asleep, or early awakening. To meet diagnostic criteria for insomnia, these difficulties must persist for at least three months and result in severe daytime impairment. Insomnia is common in menopausal women and often persists after menopause. Cognitive behavioral therapy for insomnia (CBT-I) is a concise form of behavioral therapy that has demonstrated its efficacy across the adult lifespan, including during midlife, for many years [15].

A meta-analysis conducted in 2015, encompassing 20 randomized controlled trials focused on cognitive behavioral therapy for insomnia (CBT-I) in individuals suffering from chronic insomnia, revealed an average decrease in sleep latency of 19 min post-sleep onset and a reduction in wake time averaging 26 min. Additionally, total sleep time was reduced by 8 min, while sleep efficiency improved by 10%. In a clinical trial aimed at assessing the therapeutic efficacy of both behavioral and pharmacological treatments, whether administered separately or in combination, findings indicated that young and middle-aged patients experiencing sleep-onset insomnia could gain significantly greater advantages from CBT compared to pharmacotherapy. Consequently, CBT should be regarded as a primary intervention for chronic insomnia [16].

CBT-I produces results comparable to sleeping medication, has no side effects, has fewer relapses, and results in continued improvements in sleep long after treatment ends. Long-term improvements appear to be due to patients learning to support and promote the body’s natural sleep mechanisms. While sleeping medication can mask insomnia symptoms, CBT-I promotes the actual learning process and restores the body’s natural sleep mechanisms [17].

## 2. Materials and Methods

### 2.1. Literature Search and Selection of Studies

In order to examine the research published on this subject, we undertook a literature review utilizing a scoping review methodology [18,19] to evaluate the effectiveness of CBT-I on insomnia severity among menopausal women. This scoping review was conducted in accordance with the PRISMA-ScR guidelines. The methodology and reporting followed the recommended framework to ensure transparency, reproducibility, and comprehensiveness in the review process. A scoping review possesses distinct characteristics when compared to a systematic review. Rather than assessing the literature to address a precise question regarding a particular type of intervention (for instance, a classical CBT protocol delivered by a qualified CBT therapist), it systematically evaluates the findings, highlights variations, and identifies areas for future inquiry. While the methodology remains largely consistent with that of a systematic review, publications are not excluded based on the specific type of intervention utilized. The objectives of this review were to examine the protocols employed in randomized controlled trials (RCTs), including aspects such as the number of sessions and duration of the intervention, and their effects on the primary outcome of interest, namely insomnia. Additionally, we conducted an analysis of the outcomes from RCTs that concentrated on CBT-specific interventions in contrast to those that integrated CBT-I with supplementary treatments.

A thorough examination of published scholarly articles, media outlets, and grey literature reports was conducted to address the inquiry: “what is the effect of CBT-I in menopausal women experiencing insomnia”. This review was executed in a three-step process: (1) articulating the research question and identifying the pertinent literature; (2) selecting the relevant literature; and (3) charting, aggregating, and summarizing the findings [19].

**Figure 1:**
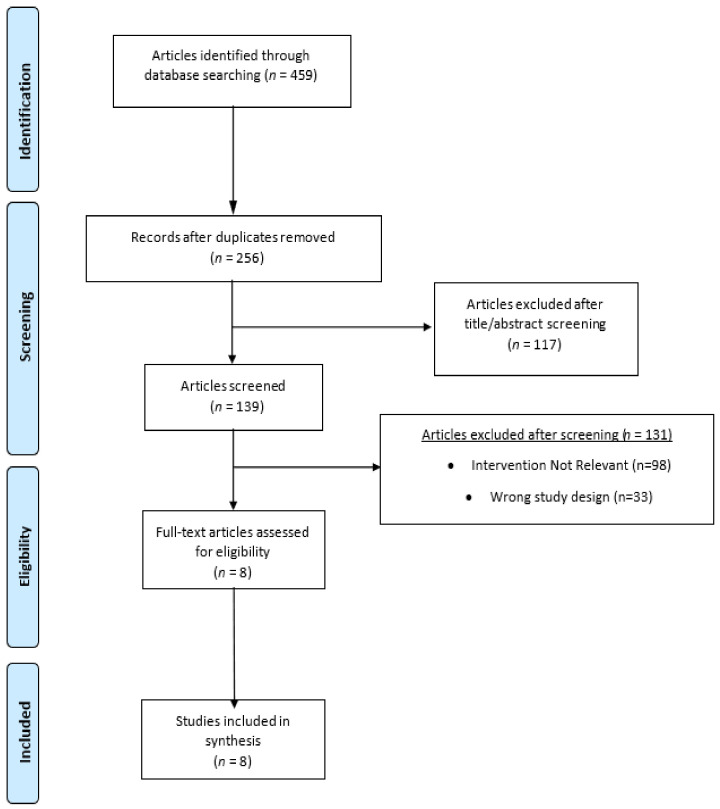
Sample, intervention, and outcomes [20].

A comprehensive search of electronic databases, including Pubmed, Scopus, Cinahl, and Medline, was undertaken. The search strategy involved the combination of medical subject headings and entry terms such as “Cognitive Behavioral Therapy”, “menopause”, and “insomnia”. This approach resulted in the identification of 459 studies, from which 8 ultimately fulfilled the criteria for inclusion (Figure 1). All publications available in the English language up to the end of July 2024 were examined. Additionally, a supplementary search was conducted using Google Scholar to verify that no pertinent papers had been overlooked. Excluded from this review were studies presented as comments, author articles, case studies, review papers, book chapters, and those lacking original data reporting.

### 2.2. Evaluation of Risks of Bias

The risk of bias in each randomized controlled study was evaluated in accordance with the Cochrane Review utilizing the NICE methodology checklist specifically designed for randomized controlled trials [21]. This checklist encompasses several domains of bias, including selection bias (pertaining to random sequence generation and allocation concealment), performance bias (involving the blinding of participants and personnel), detection bias (related to the blinding of outcome assessments), attrition bias (concerning incomplete outcome data), and reporting bias (focusing on selective reporting) [22]. The studies that met the criteria for inclusion were randomized controlled trials centered on interventions based on cognitive behavioral therapy (CBT), targeting menopausal women aged 18 years and older who had been diagnosed with insomnia [22].

## 3. Results

Our search methodology yielded a total of 459 studies. Upon the removal of duplicates, 256 papers necessitated comprehensive full-text evaluation, with 8 ultimately fulfilling the inclusion criteria and undergoing detailed analysis (Figure 1). The majority of articles were excluded due to their failure to qualify as randomized controlled trials (RCTs) or because they did not assess the primary outcome of the review. The results extracted from our literature review were independently summarized by two researchers across four categories: (1) Sample characteristics, duration of intervention, and outcomes; (2) Nature of the intervention; (3) Impact on insomnia severity; (4) Risk of bias. Conflicts between the two researchers during the screening process were likely resolved through discussion and consensus. If disagreements persisted, a third reviewer was brought in to make a final decision. Standardized criteria also helped guide the resolution process. The key data derived from the RCTs are encapsulated in Table 1 while the pertinent results associated with the review’s outcomes are detailed in the subsequent sections under the aforementioned headings.

The RCTs included have different sample sizes ranging from 46 to 546 participants diagnosed with chronic DSM-5 insomnia disorder related to menopause. All the studies were published between 2014 and 2024, with four of them performed in the USA [23,24,25,26], one in Iran [27], one in Canada [28], one in Saudi Arabia [29], and one in Republic of Korea [30].

**Table 1:** Risk of bias.

Source	Random Sequence Generation	Allocation Concealment	Blinding of Participants and Personnel	Blinding of Outcome Assessment	Incomplete Outcome Data	Selective Reporting
(Ham et al., 2020)/Republic of Korea [29]	Unclear	Unclear	High	Low	Low	Low
(Green et al., 2019)/Canada [27]	Low	Low	Unclear	Low	Low	Low
(Farsani et al., 2021)/Iran [26]	Low	High	High	Unclear	Unclear	Low
(Kalmbach et al., 2019a)/USA [23]	Unclear	Unclear	High	High	Low	Low
(McCurry et al., 2017)/USA [24]	Low	Unclear	Low	Low	Low	Low
(Drake et al., 2019)/USA [25]	Low	Low	Unclear	High	High	Low
(Guthrie et al., 2018)/USA [27]	Unclear	Unclear	High	High	Low	Low
(Abdelaziz et al., 2021)/Saudi Arabia [29]	Low	Unclear	Unclear	Unclear	Low	Low

The criteria for inclusion were largely consistent across all the randomized controlled trials (RCTs) that were considered. The eligibility requirements for participation in the studies were as follows: participants had to be postmenopausal, defined as having experienced 12 consecutive months without menstruation, report waking after sleep onset for an hour or more on three or more nights per week, and meet the criteria for insomnia disorder as outlined in the DSM-5, with either onset or worsening occurring during the peri- or postmenopausal phase.

The exclusion criteria most frequently noted in the reviewed studies encompassed prior or current major depression as defined by the DSM-5, sleep-wake disorders aside from insomnia (such as obstructive sleep apnea), and medications that affect sleep (including both prescription and non-prescription sleep aids, herbal supplements, and any antidepressants administered during nighttime), with the exception of hormone therapy.

Several RCTs employed complex randomization strategies with high-quality standards (blinding) for this type of research study [24,25,27,28]. Green and co-workers [28] conducted the blind randomization through block randomization, utilizing a computer-generated assignment sequence that was established prior to the commencement of the study. Similarly, in another RCT [27], the eligible patients were randomized using the block randomization process with a block size of four and an allocation ratio of 1:1. In the RCT by McCurry and co-workers [24], the participants were block-randomized to a CBT-I or menopause education control group. Another well-performed trial randomly allocated each participant with the use of concealed envelopes. In a separate trial, researchers compiled a comprehensive list of all participants meeting the specified criteria, subsequently assigning each individual a unique identification number. To reduce biases, random samples were selected utilizing a random number-generator software. Participants in the study were randomly allocated to either the study group or the control group, adhering to an allocation ratio of 1:1 [29]. In another study, randomization was achieved via a secure web-based database, overseen by the Data Coordinating Center, which employed a dynamic randomization algorithm [26]. The remaining trials mentioned unclear randomization strategies.

All of the randomized controlled trials (RCTs) included in the analysis featured intervention and control groups that exhibited comparable demographic characteristics, insomnia disorders associated with menopause, and initial scores for the study outcomes.

### 3.1. Type of Intervention

All randomized controlled trials (RCTs) included in this analysis assessed the impact of interventions grounded in cognitive behavioral therapy for insomnia (CBT-I), with the number of sessions typically ranging from 6 to 12 (as detailed in Table 1). Most of these trials focused on either individual or group CBT-I sessions, with session counts varying between 4 and 12 (refer to Appendix A). One trial administered internet-based CBT modules to the intervention group [29] while McCurry and co-workers administered six CBT-I or menopause education control telephone sessions in 8 weeks to the participants in the intervention group [24].

As for the main outcomes of the scoping review, the majority of the RCTs investigated the efficacy of a CBT-I program compared with regular education on how to manage insomnia. One RCT compared CBT-I for insomnia versus menopause education while three other studies compared CBT-I with sleep restriction therapy (SRT) [23,25].

The majority of the CBT-I sessions took place weekly, either with a psychologist or with a specialized nurse. In two studies, the intervention was exclusively delivered by trained registered nurses [23,25] and in another study by an experienced therapist trained by an occupational therapist [27]; and in another study, the intervention consisted of cooperation between a social worker and a psychologist that provided telephone sessions [24]. In the subsequent studies, the interventions entailed collaboration between psychologists and psychiatrists, which facilitated the provision of education on sleep hygiene [30].

This scoping review encompassed randomized clinical trials featuring either personalized or group cognitive behavioral therapy for insomnia (CBT-I) sessions. In the study conducted by Green et al., the intervention was delivered through a series of 12 weekly sessions, each lasting two hours, and conducted in a small-group format accommodating up to eight participants (with a range of 5 to 8 individuals per group). To reinforce learning, participants engaged in weekly exercises between sessions, and their progress was evaluated during the weekly group meetings. Those assigned to the waitlist condition following the baseline assessment did not partake in the CBT-Meno intervention and were instructed not to engage in any other psychological treatment throughout the 12-week waitlist duration [28]. Ham et al. conducted weekly face-to-face individual counseling sessions, providing four sessions to each woman in the experimental group. In contrast, women in the control group received a single session of group education focused on sleep hygiene and were given the same counseling booklet as their counterparts in the experimental group [30].

In a separate investigation, participants undergoing cognitive behavioral therapy for insomnia (CBT-I) attended six weekly sessions, each lasting 60 min, facilitated by a qualified therapist (HMF). Conversely, individuals in the control group were provided with standard care from menopause clinics, which included general guidance on sleep hygiene and managing complications associated with menopause [27].

In the study of Kalmbach et al. [23], women assigned to receive cognitive behavioral therapy for insomnia (CBT-I) participated in six weekly sessions that included both behavioral components, such as sleep restriction and stimulus control, and cognitive elements, including cognitive restructuring. Additionally, the program incorporated relaxation techniques, such as progressive muscle relaxation and autogenic training, along with education on sleep hygiene. In a separate study, both the CBT-I and the Medication Education Control (MEC) interventions comprised six telephone sessions lasting 20 to 30 min each, spread over an eight-week period (specifically during weeks 1 to 4, 6, and 8). While participants were given the option to attend their initial session in person at a research office, women had the flexibility to conduct Session 1 via phone if they preferred [24].

In the study of Abdelaziz and co-workers [29], the intervention group engaged in cognitive behavioral therapy (CBT) through a series of six online modules. Each module included reflections and feedback from the preceding module, a PowerPoint presentation outlining scheduled topics, instructions from the researchers, homework assignments, and videos demonstrating the application of the suggested practical skills. To elucidate the homework tasks, both oral instructions and written texts were provided. Participants received weekly feedback through WhatsApp or email. The CBT-I group participated in six weekly sessions that encompassed both behavioral components, such as sleep restriction and stimulus control, and cognitive elements, including cognitive restructuring, along with relaxation strategies, as detailed in the study conducted by Drake and colleagues [25].

In a separate investigation, female participants engaged in a telephone screening, maintained a 2-week diary documenting their sleep patterns and vasomotor symptoms (VMS), and completed a questionnaire. Those who remained eligible and submitted their consent forms were randomized and subsequently contacted via telephone by a study interventionist to arrange the initial treatment session. Participants had the option to attend their first session in person at a designated research office, though they were also allowed to conduct the initial session over the phone. A total of six telephone sessions were held over an 8-week period, with follow-up assessments occurring 8 and 24 weeks following randomization [26].

All psychological outcomes in the randomized controlled trials (RCTs) were evaluated using validated scales that possess established psychiatric indices. The majority of the studies employed the Insomnia Severity Index (ISI), a 7-item questionnaire designed to assess overall insomnia severity, as well as the Pittsburgh Sleep Quality Index (PSQI), a 19-item questionnaire aimed at evaluating sleep quality. Farsani et al. [27] and McCurry et al. [24] used the ISI, PSQI, and sleep-wake diary to collect data. Green et al. [28] utilized the Hamilton Anxiety Scale (HAM-A), which is a clinician-administered interview, to assess anxiety symptoms, and the Pittsburgh Sleep Quality Inventory (PSQI) assessed sleep difficulties over the past month with scores ranging from 0 to 21. In Ham et al. [30], the Insomnia Severity Index was employed to evaluate the extent of insomnia, while the Pittsburgh Sleep Quality Index was utilized to assess the quality of sleep.

In a separate investigation, the Insomnia Severity Index (ISI) was employed to evaluate symptoms of insomnia. Scores on the ISI of 15 or higher are indicative of clinical insomnia, whereas scores of 7 or lower suggest the absence of significant insomnia symptoms. Consequently, Guthrie et al. applied both the ISI and the Pittsburgh Sleep Quality Index (PSQI) to assess insomnia [26]. Moreover, in Drake et al. [25], in addition to the ISI, the researchers employed the Ford Insomnia Response to Stress Test (FIRST) to assess pretreatment sleep reactivity within the study population.

### 3.2. Effects on Insomnia Severity

The primary result featured in this scoping review evaluated the impact of CBT-I on the severity of insomnia in menopausal women. A majority of the randomized controlled trials indicated a notable effect on the improvement of insomnia within the intervention groups when compared to those undergoing sleep restriction therapy and standard care. Abdelaziz et al. [29] found that Internet-based cognitive behavioral therapy for insomnia (CBT-I) effectively alleviates sleep-related issues, particularly evidenced by improvements in sleep quality scores (−3.60 ± 2.76) and insomnia index scores (−5.10 ± 3.54) from the baseline measurements. Furthermore, the program resulted in notable alterations in various sleep parameters, including an increase in total sleep hours (t = 2.734, *p* = 0.008), enhanced sleep efficiency of ≥85% (t = 3.558, *p* = 0.001), and a reduction in sleep latency (t = 2.180, *p* = 0.033) when compared to the control group. In a separate randomized controlled trial (RCT), notable enhancements were observed in cognitive behavioral therapy for menopausal symptoms (CBT-Meno) when compared to the waitlist group, specifically regarding vasomotor symptom interference (HFRDIS; *p* < 0.001, *p* = 0.21) and sleep difficulties (PSQI; *p* = 0.001, *p* = 0.17). These improvements were sustained three months following the conclusion of treatment [28]. McCurry et al. [24] assessed the effectiveness of telephone-based cognitive behavioral therapy for insomnia (CBT-I) in comparison to menopause education control (MEC). After 8 weeks, participants receiving CBT-I, consisting of 53 women with a mean age of 55.0 years (SD, 3.5), exhibited a reduction of 9.9 points in Insomnia Severity Index (ISI) scores. In contrast, the MEC group, also comprising 53 women with a mean age of 54.7 years (SD, 4.7), demonstrated a decrease of 4.7 points. This resulted in a mean between-group difference of 5.2 points (95% CI, −6.1 to −3.3; *p* < 0.01). Furthermore, Pittsburgh Sleep Quality Index scores reflected a decline of 4.0 points in the CBT-I group versus 1.4 points in the MEC group, yielding a mean between-group difference of 2.7 points (95% CI, −3.9 to −1.5; *p* < 0.01). Notably, significant differences between the groups were maintained at the 24-week mark. At both 8 and 24 weeks, 33 of 47 women (70%) and 37 of 44 women (84%) in the CBT-I group achieved ISI scores indicating no insomnia, compared to only 10 of 41 women (24%) and 16 of 37 women (43%) in the MEC group, respectively. Additionally, the CBT-I group reported greater improvements in sleep latency, wake time, and sleep efficiency as recorded in diaries. While there were no significant differences between the groups regarding the frequency of daily hot flashes, the CBT-I group experienced a notable reduction in hot flash interference at 8 weeks (−15.7; 95% CI, −20.4 to −11.0) compared to the MEC group (−7.1; 95% CI, −14.6 to 0.4) (*p* = 0.03), with these differences persisting at 24 weeks for the CBT-I group (−22.8; 95% CI, −28.6 to −16.9) and MEC group (−11.6; 95% CI, −19.4 to −3.8) (*p* = 0.003).

In Kalmbach et al. [23], the findings indicated that both cognitive behavioral therapy for insomnia (CBTI) and Sleep Restriction Therapy (SRT) yielded moderate to large enhancements in fatigue, energy levels, sleepiness, and work functioning, observed at the conclusion of treatment and maintained six months thereafter. Participants in the CBTI group experienced a notable improvement in quality of life, as evidenced by significant gains in emotional well-being and resilience to both physical and emotional challenges. In contrast, the SRT and Sleep Hygiene Education (SHE) groups exhibited improvements solely in resilience to physical issues. As sleep quality improved, there was a reduction in pain complaints; however, these changes were not linked to any particular treatment modality. Likewise, individuals who remitted from insomnia reported fewer occurrences of hot flashes during both daytime and nighttime, although these reductions were not tied to any specific treatment approach. In the study of Drake et al. [25], from baseline to post-treatment, the ISI exhibited a reduction of 7.70 points within the CBTI group (*p* < 0.001), a decrease of 6.56 points in the SRT group (*p* < 0.001), and a decline of 1.12 points in the SHE group (*p* = 0.01). While all groups experienced an increase in average sleep duration by the six-month follow-up, patients undergoing CBTI achieved an additional 40 to 43 min of sleep per night compared to those receiving SHE or SRT. At both post-treatment and the six-month follow-up, remission rates were significantly higher in the CBTI (54–84%) and SRT (38–57%) groups than in the SHE patients (4–33%). Generally, CBTI patients demonstrated a greater likelihood of achieving remission compared to SRT patients.

In the study of Ham et al. [30], the findings suggested that four sessions of individual counseling, along with education on sleep hygiene, effectively enhanced both insomnia and overall sleep quality. The results from repeated measures MANOVA indicated a significant main effect of time on anthropometric variables, including body mass index, waist circumference, and blood pressure. Additionally, significant main effects of both group and time were observed concerning psychosocial variables such as sleep quality, insomnia, depressive symptoms, and quality of life (*p* < 0.05). Furthermore, results from the repeated measures ANOVA demonstrated a significant impact of the group on insomnia and sleep quality (*p* < 0.05). In summary, the intervention proved to be effective in addressing insomnia and improving sleep quality.

In another RCT [27], in the CBT-I group, a continuous reduction in the mean scores for ISI, PSQI, sleep onset latency, sleep duration, and sleep quality, was observed from baseline to week 3, as well as from week 3 to week 6, with these scores remaining stable from week 6 to week 10 when compared to the control group. Additionally, the mean sleep efficiency score exhibited a significant improvement from baseline to week 3 in the CBT-I group, maintaining this level of enhancement until the conclusion of the study.

In the study of Guthrie et al. [26], CBT-I yielded significantly greater alleviation of insomnia symptoms compared to the other active interventions. In an adjusted model, the average reduction in the Insomnia Severity Index (ISI) from baseline to follow-up for the CBT-I group was 5.2 points relative to the control group. Furthermore, the influence of CBT-I on the Pittsburgh Sleep Quality Index (PSQI) was markedly more pronounced than that of the other interventions, with a mean PSQI reduction of 2.7 points from baseline to follow-up in the CBT-I group relative to control.

### 3.3. Risk of Bias

We used the previously reported Cochrane guidelines [21] to separately analyze each type of bias in each of the eight trials we included. A summary is shown in Table 1. Typically, the patients, psychologists, nurses, and/or social workers participating in the trial could not be blinded to the intervention group, as the specifics of the programs were generally outlined in the informed consent. It is important to note that in randomized controlled trials (RCTs), blinding differs from allocation concealment. The concept of “allocation concealment” serves to mitigate bias by obscuring the assignment of study participants to treatment groups, thereby preventing any potential exploitation of this information. Proper allocation concealment is essential in preventing study participants from affecting the assignment of treatments to subjects [22]. Research that lacks adequate allocation concealment, or exhibits deficiencies in this regard, is vulnerable to selection bias. In this regard, the risk of bias for two [25,28] of the RCTs was limited due to the fact that the individual administering the CBT-I intervention remained unaware of the treatment condition and timing during all assessments. Furthermore, five of the studies included exhibited unclear allocation concealment, rendering them susceptible to selection bias. Two randomized controlled trials did not provide adequate detail regarding the methodology employed to conceal the allocation sequence [26,27]. With regard to random sequence generation, the randomization procedure was unclear in three trials [23,26,30] and adequate in the remaining ones.

Performance bias such as blinding of the participants and personnel was clearly met in the study of McCurry et al. [24], while the remaining studies had unclear [25,28] or high [23,27] risk of bias for this criterion. Blinding of outcome assessment was adequate in three trials [24,28,30] and unclear in one [27], while a high risk of bias was detected in the remaining three studies [23,25,26]. The presence of attrition bias resulting from the collection of incomplete outcomes or selective reporting data was generally low in five trials or unclear in one trial [27], whereas there was a high risk of bias in the study of Drake et al. [25] regarding the reporting bias; all the included trials presented a low risk of bias.

## 4. Discussion

This scoping review examined the impact of CBT-I interventions on the severity of insomnia among menopausal women. During the transition to menopause, women often encounter sleep disturbances, notably experiencing night-time awakenings. The outcome measures derived from the randomized controlled trials (RCTs) encompassed insomnia symptoms, overall sleep quality, and quality of life. All studies included in the review indicated a significant effect of the CBT-I intervention on both sleep quality and insomnia symptoms in the intervention groups when compared to the control group. These findings align with the meta-analysis conducted by Lam et al. [31], which demonstrated through subgroup analyses that both cognitive behavioral therapy and mindfulness/relaxation techniques yielded improvements in sleep, assessed via subjective measures such as the Pittsburgh Sleep Quality Index, as well as objective metrics. The authors determined that behavioral interventions—particularly cognitive behavioral therapy, physical exercise, and mindfulness/relaxation—serve as effective treatments for enhancing sleep outcomes in perimenopausal and postmenopausal women. In their systematic review, Williams et al. [32] identified that cognitive behavioral therapy for insomnia (CBT-I) serves as an effective intervention for co-morbid insomnia. This treatment modality is especially appealing as it does not entail certain risks commonly associated with pharmacological approaches, such as dependency, polypharmacy, and impairments in cognitive and psychomotor functioning.

A number of meticulously designed studies offer initial evidence indicating that cognitive behavioral therapy for insomnia (CBT-I) significantly enhances insomnia parameters in menopausal women, while findings from rigorously conducted randomized controlled trials (RCTs) further endorse the application of Sleep Restriction Therapy (SRT). Compared with MEC and SHE, both treatments demonstrated similar durable and clinically meaningful effects on insomnia severity, sleep quality, and various sleep disorder measures [24,25]. Moreover, both CBT-I and SRT demonstrated comparable effects on various secondary outcomes, such as depression, daytime symptoms, and work performance. While both treatments yielded enhancements in these domains, CBT-I emerged as the more effective option due to its provision of more immediate, sustained, and substantial reductions in depression, dysfunctional sleep beliefs, and presleep somatic hyperarousal. Nevertheless, the treatment effects on overall cognitive arousal—including worry, rumination, and presleep perseverative thoughts—were found to be less pronounced. This indicates that postmenopausal women experiencing insomnia may benefit from refining CBT-I and SRT to address cognitive arousal and stress dysregulation more effectively, potentially enhancing treatment outcomes for both insomnia and depression, while also decreasing the likelihood of future relapse of these conditions [23]. Furthermore, a comprehensive analysis of the MsFLASH trial indicates that cognitive behavioral therapy for insomnia (CBT-I) outperforms various pharmacological and nonpharmacological interventions in women experiencing HFNS issues along with clinically significant symptoms of insomnia [26]. Clinical trial data consistently indicate that cognitive behavioral therapy for insomnia (CBTI) is equally as effective as pharmacological interventions in the short term, yet it yields a more favorable treatment response over the long term [33,34].

Furthermore, the studies analyzed encompassed various approaches to administering psychological interventions, which included differences in treatment duration ranging from 4 to 8 weeks, modes of delivery (such as face-to-face versus telehealth interventions), and the diverse therapists who facilitated the interventions. Consequently, these differences underscore the variability inherent in these interventions. For example, prior research has indicated that a significant obstacle to obtaining insomnia treatment is the shortage of healthcare providers trained in cognitive behavioral therapy for insomnia (CBT-I) [35].

The Insomnia Severity Index (ISI) and the Pittsburgh Sleep Quality Index (PSQI) are the most commonly utilized scales for assessing the severity of insomnia symptoms. There exists a variety of patient-reported questionnaires designed to evaluate insomnia symptoms, their intensity, associated factors, and various elements believed to play a role in the development of insomnia [36,37]. In terms of screening and assessing insomnia, there are fewer tools available to evaluate treatment outcomes. Notably, the Insomnia Severity Index [38], Pittsburgh Sleep Quality Index [39], Insomnia Symptom Questionnaire [40], and Athens Insomnia Scale [41] are among the most frequently employed instruments for these purposes. Time frames vary between instruments, and they are generally designed to assess patient perceptions and quantify subjective levels of insomnia. The Insomnia Severity Index (ISI) is a brief tool designed to assess the severity of nighttime and daytime insomnia. It is available in many languages and is increasingly used in clinical research as a measure of response to treatment. Its psychometric properties have been previously documented using classical test theory [38,42,43,44,45].

The Pittsburgh Sleep Quality Index (PSQI) serves as a widely recognized instrument for assessing sleep disturbances and has been utilized across various populations [45,46]. Comprising 19 items, it generates a total score that enables researchers to evaluate sleep dysfunction over a span of 10 months [38]. In the investigation conducted by McCurry et al. [24], ISI scores exhibited a reduction of 9.9 points among 53 women undergoing CBT-I and a decrease of 4.7 points among 53 women receiving MEC, resulting in an average between-group difference of 5.2 points. Additionally, PSQI scores diminished by 4.0 points in the CBT-I group and by 1.4 points in the MEC group, yielding a mean between-group difference of 2.7 points. Notably, significant group differences persisted at the 24-week mark. In a separate randomized controlled trial (RCT) [25], ISI scores declined by 7.70 points in the CBT-I cohort (*p* < 0.001), by 6.56 points in the SRT cohort (*p* < 0.001), and by 1.12 points in the SHE cohort (*p* = 0.01).

Nevertheless, several limitations must be considered prior to generalizing these findings to the broader population. Notably, the heterogeneity in selection criteria and the varying quality of the studies related to randomization processes and participant blinding represent significant limitations observed in the included research. Additionally, the interventions applied in the control groups differed across studies; for instance, McCurry et al. [24] compared the efficacy of telephone-based CBT-I in the intervention group against menopause education in the control group. In contrast, two other randomized controlled trials (RCTs) [23,24,25] assessed sleep restriction therapy against the CBT-I intervention, while the remaining studies provided participants in the control groups with routine care at menopause clinics, which included general information on sleep hygiene and managing menopause-related complications. Furthermore, discrepancies existed among the samples included in the RCTs, as most comprised a small number of participants who were predominantly white and well-educated females, thereby limiting the applicability of the results to minority women or those from lower educational backgrounds. Numerous studies also excluded women with depression, those with moderate to severe depressive symptoms, and those taking psychotropic medications. Future investigations should focus on evaluating the effectiveness of these interventions in women experiencing clinical depression [47,48,49].

The limitations inherent in this scoping review encompass the heterogeneity present within the sample, characterized by a considerable variation in participant numbers, as well as the relatively brief follow-up duration observed in the majority of the included studies. Consequently, this indicates that additional variables, including patient characteristics and the duration of interventions, may significantly influence both the robustness of the findings and their sustainability over time during follow-up. Future randomized controlled trials (RCTs) should investigate the effects of cognitive behavioral therapy for insomnia (CBT-I) in relation to the severity of insomnia symptoms.

The findings of our study further establish the efficacy of cognitive behavioral therapy for insomnia (CBT-I) interventions in addressing insomnia symptoms among postmenopausal women. The evidence supports the effectiveness of CBT-I for insomnia linked to menopause and indicates its potential role in alleviating concurrent depressive symptoms. Additionally, improvements in insomnia symptoms seem to be maintained for up to six months after the completion of CBT-I and may continue to diminish over time. Consequently, CBT-I has the potential to significantly enhance sleep quality and reduce the severity of insomnia in postmenopausal women. Therefore, it is advisable to utilize this therapeutic approach for menopausal women experiencing insomnia.

## Appendix A

**Table A1:** Included studies.

References Study/Country Where the RCT Was Performed	Number of Patients, Mean Age, and Allocation	Design and Intervention	Outcomes
McCurry et al., 2016 USA [24]	A total of 106 participants (mean [SD] age, 54.8 [4.2] years) were randomly assigned to the 2 intervention arms. 53 Randomized to CBT-I, 53 Randomized to MEC	Over the course of eight weeks, participants engaged in six telephone sessions, each lasting between 20 and 30 min, focused on either CBT-I or Menopause Education Control (MEC). Weekly electronic sleep diaries were submitted by the participants, who also received written educational materials tailored to their specific group. The CBT-I sessions encompassed techniques such as sleep restriction, stimulus control, education on sleep hygiene, cognitive restructuring, and the assignment of behavioral homework, while the MEC sessions delivered information pertaining to menopause and women’s health.	At the 8-week mark, a reduction of 9.9 points in ISI scores was observed among 53 women undergoing CBT-I (mean [SD] age, 55.0 [3.5] years), while a decrease of 4.7 points was noted among 53 women receiving MEC (mean age, 54.7 [4.7] years). This resulted in an average between-group difference of 5.2 points (95% CI, −6.1 to −3.3; *p* < 0.001). Additionally, scores on the Pittsburgh Sleep Quality Index declined by 4.0 points for those in the CBT-I group and by 1.4 points for those in the MEC group, leading to a mean difference of 2.7 points (95% CI, −3.9 to −1.5; *p* < 0.001). Notably, significant differences between groups persisted at the 24-week follow-up. At both the 8-week and 24-week evaluations, 33 of 47 women (70%) and 37 of 44 women (84%) in the CBT-I group demonstrated ISI scores within the no-insomnia range, in contrast to 10 of 41 women (24%) and 16 of 37 women (43%) in the MEC group, respectively. Furthermore, the CBT-I group exhibited more substantial improvements in diary-reported metrics such as sleep latency, wake time, and sleep efficiency. While there were no significant differences between the groups regarding the frequency of daily hot flashes, the CBT-I group experienced a notable reduction in hot flash interference at 8 weeks (−15.7; 95% CI, −20.4 to −11.0) compared to the MEC group (−7.1; 95% CI, −14.6 to 0.4) (*p* = 0.03). These differences were maintained at 24 weeks, with the CBT-I group showing a reduction of −22.8 (95% CI, −28.6 to −16.9) and the MEC group a reduction of −11.6 (95% CI, −19.4 to −3.8) (*p* = 0.003).
Guthrie et al., 2018 USA [26]	546 peri- and postmenopausal women with Insomnia Severity Index (ISI) ≥ 12, and ≥14 bothersome VMS/week across the four RCTs	The interventions administered comprised the following: escitalopram at a dosage of 10–20 mg per day; yoga practices; aerobic physical activity; omega-3 fatty acids at a daily intake of 1.8 g; oral administration of 17-beta-estradiol at 0.5 mg per day; venlafaxine XR at a dosage of 75 mg per day; and cognitive behavioral therapy for insomnia (CBT-I). The outcome measures evaluated included the Insomnia Severity Index (ISI) and the Pittsburgh Sleep Quality Index (PSQI) over a treatment duration of 8 to 12 weeks.	Cognitive behavioral therapy for insomnia (CBT-I) demonstrated the most substantial decrease in the Insomnia Severity Index (ISI) from baseline compared to the control group, achieving a reduction of −5.2 points (95% CI −7.0 to −3.4). The effects on ISI were similarly observed with exercise, which recorded a decrease of −2.1 points, and with venlafaxine, which showed a reduction of −2.3 points. Minor reductions in ISI were also noted with escitalopram, yoga, and estradiol. The most significant decline in the Pittsburgh Sleep Quality Index (PSQI) from baseline was associated with CBT-I, yielding a reduction of −2.7 points (−3.9 to −1.5). However, decreases in PSQI ranging from 1.2 to 1.6 points were significantly superior to control for escitalopram, exercise, yoga, estradiol, and venlafaxine. No improvement in insomnia symptoms was observed with omega-3 supplements.
Green et al., 2019 Canada [28]	A total of 72 participants (Mage = 53.08, SD = 4.02, Range = 43–62) were randomly allocated to either a 12-week CBT-Meno intervention group (n = 37) or a 12-week waitlist control group (n = 35).	CBT-Meno was conducted in a small-group format consisting of 12 sessions, each lasting 2 h, with a maximum of eight participants per group (ranging from 5 to 8 individuals). To reinforce learning, participants engaged in weekly exercises between sessions, and their progress was evaluated during the weekly group meetings. Those assigned to the waitlist condition following the baseline assessment did not participate in the CBT-Meno intervention and were prohibited from receiving any alternative psychological treatment throughout the 12-week waitlist duration. A re-assessment of all waitlist participants occurred at the conclusion of the 12 weeks post-baseline. Subsequently, these waitlist participants were provided the opportunity to undergo the CBT-Meno treatment after completing the 12-week post-baseline evaluation.	Significant enhancements were observed in CBT-Meno compared to the waitlist group regarding vasomotor symptom interference (HFRDIS; *p* < 0.001), levels of “bothersomeness” (GCS-vm; *p* = 0.04), depressive symptoms (BDI-II; *p* = 0.001), sleep difficulties (PSQI; *p* = 0.001), and sexual concerns (GCS-sex; *p* = 0.03). These findings remained consistent even after accounting for menopausal staging and medication usage. Improvements were sustained at three months following the conclusion of treatment.
Kalmbach et al., 2019 USA [23]	A total of 117 postmenopausal women (average age 56.34 ± 5.41 years) experiencing chronic insomnia with either peri- or postmenopausal onset were randomly assigned to one of three treatment conditions: a control group receiving sleep hygiene education (SHE), Sleep Restriction Therapy, and cognitive behavioral therapy for insomnia (CBTI). Assessments conducted in a blinded manner were carried out at baseline, following treatment, and at a 6-month follow-up.	Sleep hygiene education (SHE), serving as a minimal intervention control condition, involved women who were assigned to the online sleep hygiene education group receiving six weekly emails. These communications contained general, non-personalized information covering various topics, including fundamental principles of endogenous sleep regulation; the influence of sleep on health issues such as obesity, diabetes, and hypertension; the effects of stimulants and other substances that disrupt sleep; the interplay between sleep, diet, and physical activity; as well as recommendations for establishing an environment conducive to restful sleep. The SRT intervention was conducted over a period of two weeks. The first session, which took place in person, involved an examination of the patient’s sleep history, along with education regarding the rationale behind sleep restriction practices and the assignment of behavioral homework. Subsequently, four follow-up sessions were arranged over the next two weeks, consisting of three phone contacts spaced 3 to 4 days apart, culminating in a second face-to-face session. These follow-up sessions were utilized to adjust sleep schedules in accordance with data obtained from sleep diaries. Patients undergoing Cognitive Behavioral Therapy for Insomnia (CBTI) participated in six weekly sessions that encompassed both behavioral components—such as sleep restriction and stimulus control—and cognitive elements, including cognitive restructuring. Additionally, the program incorporated relaxation techniques, such as progressive muscle relaxation and autogenic training, alongside education on sleep hygiene. To ensure fidelity in the therapeutic process, nurse therapists engaged in weekly supervision meetings with one of two licensed PhD clinical psychologists, both of whom hold certification in behavioral sleep medicine. These supervision sessions involved case discussions, problem-solving activities, and the review of recorded therapy sessions, allowing for constructive feedback.	Cognitive behavioral therapy for insomnia (CBTI) resulted in moderate to substantial decreases in depressive symptoms, while Sleep Restriction Therapy (SRT) exhibited moderate reductions, albeit not until six months following treatment. The effects of these treatments on maladaptive cognition presented a mixed picture. Both CBTI and SRT achieved significant advancements in addressing dysfunctional beliefs regarding sleep; however, their impact on pre-sleep cognitive arousal, rumination, and worry was less pronounced. Notably, pre-sleep somatic arousal saw considerable improvement within the CBTI cohort and moderate enhancement in the SRT cohort. The observed improvements in depression, maladaptive thinking, and hyperarousal were associated with enhancements in sleep quality. In contrast, SHE demonstrated no lasting treatment effects.
Drake et al., 2019 [25]	A total of 150 postmenopausal women, aged 56.44 ± 5.64 years, diagnosed with chronic insomnia disorder as per DSM-5 criteria associated with menopause, were randomly assigned to one of three treatment modalities: sleep hygiene education (SHE), stimulus response therapy (SRT), or cognitive behavioral therapy for insomnia (CBTI).	Women participating in cognitive-behavioral therapy for insomnia were randomly assigned to undergo six in-person sleep therapy sessions with a registered nurse who was an expert in behavioral sleep medicine. SRT was implemented as an intervention spanning a duration of two weeks. Specifically, the initial in-person session involved a comprehensive review of the patient’s sleep history, alongside an educational component that outlined the rationale for sleep restriction practices and the assignment of behavioral homework. This session was conducted by a registered nurse with expertise in behavioral sleep medicine. Subsequently, four follow-up sessions were held, three of which… Phone consultations, scheduled every 3 to 4 days, were conducted over the subsequent two weeks, culminating in a second face-to-face meeting. These interactions were utilized to adjust sleep schedules in accordance with the data recorded in sleep diaries. Participants in the online SHE condition, who were randomly assigned, received a series of six weekly emails containing general, non-personalized information covering various subjects: the fundamentals of endogenous sleep regulation; the influence of sleep on health issues including obesity, diabetes, and hypertension; the effects of stimulants and other substances that disrupt sleep; the interconnections among sleep, diet, and exercise; as well as recommendations for establishing a bedroom environment conducive to sleep.	From the baseline to the posttreatment phase, a reduction of 7.70 points in the ISI was observed in the CBTI group (*p* < 0.001), while the SRT group experienced a decrease of 6.56 points (*p* < 0.001), and the SHE group showed a decline of 1.12 points (*p* = 0.01). At the six-month follow-up, all groups reported an increase in average sleep duration; however, patients in the CBTI group achieved 40 to 43 more minutes of sleep each night compared to their counterparts. Patients who underwent SHE or SRT exhibited varying remission rates. At posttreatment and the 6-month follow-up, the CBTI group showed remission rates ranging from 54% to 84%, while the SRT group had rates between 38% and 57%. In contrast, patients receiving SHE demonstrated significantly lower remission rates, ranging from 4% to 33%. Overall, individuals in the CBTI group were more likely to achieve remission compared to those in the SRT group.
Ham et al., 2020 Republic of Korea [30]	In the CBT-I group, there were 24 participants with a mean age of 53.83 ± 6.64, while the control group comprised 20 individuals with a mean age of 55.45 ± 4.43.	The CBT-I intervention included a single group session followed by four individual sessions. The group session, which focused on sleep hygiene education, took place in a lecture hall situated within the PPFK branch office prior to the commencement of individual counseling. A sleep psychologist and psychiatrist delivered a two-hour session on sleep hygiene education. This educational content encompassed the causes, signs, and symptoms of insomnia, as well as activities that may either enhance or detract from sleep quality. Additionally, it addressed the characteristics of an environment conducive to sleep and clarified common misconceptions regarding sleep and sleeping medications. For each woman in the experimental group, four weekly sessions of individual counseling were offered in a face-to-face format. Two nurse counselors, who had received training, conducted the individual counseling sessions. The interveners were trained by a nurse practitioner specializing in sleep research.	The findings of the study demonstrated that four sessions of individual counseling, when paired with education on sleep hygiene, proved to be effective in enhancing both insomnia and sleep quality. In summary, the intervention successfully contributed to the improvement of insomnia and inadequate sleep quality.
Moradi Farsani et al., 2021 Iran [27]	CBT-I group (n = 22) with a mean age of 51.41 (3.00, 46–56), Control group (n = 23) with a mean age of 52.35 (3.48, 46–57)	This study was conducted as a randomized clinical trial involving the recruitment of 46 women who were randomly assigned to one of two groups: one group received cognitive behavioral therapy for insomnia (CBT-I), while the other served as a control group. Participants in the CBT-I group underwent six sessions of training, whereas those in the control group received standard care only.	In comparison to the control group, the CBT-I group exhibited a continuous reduction in the mean scores for ISI, PSQI, sleep onset latency, sleep duration, and sleep quality from baseline to week 3, as well as from week 3 to week 6. These scores remained stable from week 6 to week 10. Furthermore, the mean sleep efficiency score demonstrated a significant improvement from baseline to week 3 within the CBT-I group, maintaining this enhancement until the conclusion of the study. The findings indicate that CBT-I can lead to notable improvements in both insomnia severity and sleep quality among postmenopausal women.
Abdelaziz et al., 2021 [29]	80 menopausal women; The mean age was 53.06 ± 4.28 years	The group receiving the intervention underwent cognitive behavioral therapy (CBT) through a series of six online modules. Each module included reflections and feedback from the participants. In the preceding module, a PowerPoint presentation was utilized to organize topics, provide researchers’ guidelines, outline homework assignments, and present videos pertaining to the application of the suggested practical skills. Additionally, oral instructions were given. Textual materials were supplied to elucidate the homework assignments. Participants were provided with weekly feedback through WhatsApp or email. The material for each module was presented on a weekly basis at a consistent, predetermined time. Control intervention: Interaction between researchers and participants was minimal. The researchers addressed the concerns and needs of the participants without engaging in any form of intervention.	Internet-based cognitive behavioral therapy (CBT) proved effective in alleviating sleep difficulties, notably evidenced by reductions in sleep quality scores (−3.60 ± 2.76) and insomnia index scores (−5.10 ± 3.54) from baseline measurements. Furthermore, the program facilitated substantial alterations in various sleep parameters. Notable outcomes include an increase in total sleep duration (t = 2.734, *p* = 0.008), an enhancement in sleep efficiency of 85% or greater (t = 3.558, *p* = 0.001), and a reduction in sleep latency (t = 2.180, *p* = 0.033) when compared to the control group.

## References

[1] Daley A.J., Thomas A., Roalfe A.K., Stokes-Lampard H., Coleman S., Rees M., Hunter M.S., MacArthur C. (2015). The Effectiveness of Exercise as Treatment for Vasomotor Menopausal Symptoms: Randomised Controlled Trial. BJOG.

[2] Guidozzi F. (2013). Sleep and Sleep Disorders in Menopausal Women. Climacteric.

[3] Zhang J.P., Wang Y.Q., Yan M.Q., Li Z.A., Du X.P., Wu X.Q. (2016). Menopausal Symptoms and Sleep Quality During Menopausal Transition and Postmenopause. Chin. Med. J..

[4] Baker F.C., Lampio L., Saaresranta T., Polo-Kantola P., Kalleinen N., Vahlberg T., Polo O. (2018). Sleep and Sleep Disorders in the Menopausal Transition. Sleep Med. Clin..

[5] Xu Q., Lang C.P., Rooney N. (2014). A Systematic Review of the Longitudinal Relationships Between Subjective Sleep Disturbance and Menopausal Stage. Maturitas.

[6] Bruyneel M. (2015). Sleep Disturbances in Menopausal Women: Aetiology and Practical Aspects. Maturitas.

[7] Gold E.B., Sternfeld B., Kelsey J.L., Brown C., Mouton C., Reame N., Salamone L. (2000). Relation of Demographic and Lifestyle Factors to Symptoms in a Multi-Racial/Ethnic Population of Women 40–55 Years of Age. Am. J. Epidemiol..

[8] Joffe H., Massler A., Sharkey K.M. (2010). Evaluation and Management of Sleep Disturbance During the Menopause Transition. Semin. Reprod. Med..

[9] Kravitz H.M., Joffe H. (2011). Sleep During the Perimenopause: A SWAN Story. Obstet. Gynecol. Clin. N. Am..

[10] Wesstrom J., Nilsson S., Sundstrom-Poromaa I., Lindberg E., Ekberg N., Linde E. (2008). Restless Legs Syndrome Among Women: Prevalence, Co-Morbidity and Possible Relationship to Menopause. Climacteric.

[11] Galvan T., Camuso J., Sullivan K., Boselli M., Tonnu C., Drake C.L. (2017). Association of Estradiol with Sleep Apnea in Depressed Perimenopausal and Postmenopausal Women: A Preliminary Study. Menopause.

[12] Zolfaghari S., Yao C., Thompson C., Rej S., Battersby S., Richardson S. (2019). Effects of Menopause on Sleep Quality and Sleep Disorders. Canadian Longitudinal Study on Aging. Menopause.

[13] Kravitz H.M., Ganz P.A., Bromberger J., Powell L.H., Sutton-Tyrrell K., Meyer P.M. (2003). Sleep Difficulty in Women at Midlife: A Community Survey of Sleep and the Menopausal Transition. Menopause.

[14] El Khoudary S.R., Greendale G., Crawford S.L., Avis N.E., Brooks M.M., Thurston R.C., Sternfeld B., Sutton-Tyrrell K., Karvonen-Gutierrez C.A., Powell L.H. (2019). The Menopause Transition and Women’s Health at Midlife: A Progress Report from the Study of Women’s Health Across the Nation (SWAN). Menopause.

[15] Lam C.M., Hernandez-Galan L., Mbuagbaw L., Ewusie J.E., Thabane L., Shea A.K. (2022). Behavioral Interventions for Improving Sleep Outcomes in Menopausal Women: A Systematic Review and Meta-Analysis. Menopause.

[16] Jacobs G.D., Pace-Schott E.F., Stickgold R., Otto M.W. (2004). Cognitive-Behavior Therapy and Pharmacotherapy for Insomnia: A Randomized Controlled Trial and Direct Comparison. Arch. Intern. Med..

[17] Trauer J.M., Qian M.Y., Doyle J.S., Rajaratnam S.M., Cunnington D. (2015). Cognitive Behavioral Therapy for Chronic Insomnia: A Systematic Review and Meta-Analysis. Ann. Intern. Med..

[18] Munn Z., Peters M.D.J., Stern C., Tufanaru C., McArthur A., Aromataris E. (2018). Systematic Review or Scoping Review? Guidance for Authors When Choosing Between a Systematic or Scoping Review Approach. BMC Med. Res. Methodol..

[19] Arksey H., O’Malley L. (2005). Scoping Studies: Towards a Methodological Framework. Int. J. Soc. Res. Methodol. Theory Pract..

[20] Moher D., Liberati A., Tetzlaff J., Altman D.G., The PRISMA Group (2009). Preferred Reporting Items for Systematic Reviews and Meta-Analyses: The PRISMA Statement. PLoS Med..

[21] Higgins J.P.T., Altman D.G., Gøtzsche P.C., Jüni P., Moher D., Oxman A.D., Savović J., Schulz K.F., Weeks L., Sterne J.A.C. (2011). The Cochrane Collaboration’s Tool for Assessing Risk of Bias in Randomised Trials. BMJ.

[22] (2012). Appendix C: Methodology Checklist: Randomised Controlled Trials|Tools and Resources|The Guidelines Manual|Guidance|NICE. https://www.nice.org.uk/process/pmg6/resources/the-guidelines-manual-appendices-bi-2549703709/chapter/appendix-c-methodology-checklist-randomised-controlled-trials.

[23] Kalmbach D.A., Cheng P., Arnedt J.T., Anderson J.R., Roth T., Fellman-Couture C., Williams R.A., Drake C.L. (2019). Treating Insomnia Improves Depression, Maladaptive Thinking, and Hyperarousal in Postmenopausal Women: Comparing Cognitive-Behavioral Therapy for Insomnia (CBTI), Sleep Restriction Therapy, and Sleep Hygiene Education. Sleep Med..

[24] McCurry S.M., Guthrie K.A., Morin C.M., Woods N.F., Landis C.A., Ensrud K.E., Larson J.C., Joffe H., Cohen L.S., Hunt J.R. (2016). Telephone-Based Cognitive Behavioral Therapy for Insomnia in Perimenopausal and Postmenopausal Women with Vasomotor Symptoms: A MsFLASH Randomized Clinical Trial. JAMA Intern. Med..

[25] Drake C.L., Kalmbach D.A., Arnedt J.T., Cheng P., Tonnu C.V., Cuamatzi-Castelan A., Fellman-Couture C. (2019). Treating Chronic Insomnia in Postmenopausal Women: A Randomized Clinical Trial Comparing Cognitive-Behavioral Therapy for Insomnia, Sleep Restriction Therapy, and Sleep Hygiene Education. Sleep.

[26] Moradi Farsani H., Afshari P., Sadeghniiat Haghighi K., Gholamzadeh Jefreh M., Abedi P., Haghighizadeh M.H. (2021). The Effect of Group Cognitive Behavioural Therapy for Insomnia in Postmenopausal Women. J. Sleep Res..

[27] Guthrie K.A., Larson J.C., Ensrud K.E., Anderson G.L., Carpenter J.S., Freeman E.W., Joffe H., LaCroix A.Z., Manson J.E., Morin C.M. (2018). Effects of Pharmacologic and Nonpharmacologic Interventions on Insomnia Symptoms and Self-Reported Sleep Quality in Women with Hot Flashes: A Pooled Analysis of Individual Participant Data from Four MsFLASH Trials. Sleep.

[28] Green S.M., Donegan E., Frey B.N., Fedorkow D.M., Key B.L., Streiner D.L., McCabe R.E. (2019). Cognitive Behavior Therapy for Menopausal Symptoms (CBT-Meno): A Randomized Controlled Trial. Menopause.

[29] Abdelaziz E.M., Elsharkawy N.B., Mohamed S.M. (2022). Efficacy of Internet-Based Cognitive Behavioral Therapy on Sleeping Difficulties in Menopausal Women: A Randomized Controlled Trial. Perspect. Psychiatr. Care.

[30] Ham O.K., Lee B.G., Choi E., Choi S.J. (2020). Efficacy of Cognitive Behavioral Treatment for Insomnia: A Randomized Controlled Trial. West J. Nurs. Res..

[31] Williams J., Roth A., Vatthauer K., McCrae C.S. (2013). Cognitive Behavioral Treatment of Insomnia. Chest.

[32] Riemann D., Perlis M.L. (2009). The Treatments of Chronic Insomnia: A Review of Benzodiazepine Receptor Agonists and Psychological and Behavioral Therapies. Sleep Med. Rev..

[33] Qaseem A., Kansagara D., Forciea M.A., Cooke M., Denberg T.D. (2016). Management of Chronic Insomnia Disorder in Adults: A Clinical Practice Guideline from the American College of Physicians. Ann. Intern. Med..

[34] Koffel E., Bramoweth A.D., Ulmer C.S. (2018). Increasing Access to and Utilization of Cognitive Behavioral Therapy for Insomnia: A Narrative Review. J. Gen. Intern. Med..

[35] Sateia M.J., Doghramji K., Hauri P.J., Morin C.M. (2000). Evaluation of Chronic Insomnia: An American Academy of Sleep Medicine Review. Sleep.

[36] Schutte-Rodin S., Broch L., Buysse D., Dorsey C., Sateia M. (2008). Clinical Guideline for the Evaluation and Management of Chronic Insomnia in Adults. J. Clin. Sleep Med..

[37] Bastien C.H., Vallières A., Morin C.M. (2001). Validation of the Insomnia Severity Index as an Outcome Measure for Insomnia Research. Sleep Med..

[38] Buysse D.J., Reynolds C.F., Monk T.H., Berman S.R., Kupfer D.J. (1989). The Pittsburgh Sleep Quality Index: A New Instrument for Psychiatric Practice and Research. Psychiatry Res..

[39] Spielman A.J., Saskin P., Thorpy M.J. (1987). Treatment of Chronic Insomnia by Restriction of Time in Bed. Sleep.

[40] Soldatos C.R., Dikeos D.G., Paparrigopoulos T.J. (2000). Athens Insomnia Scale: Validation of an Instrument Based on ICD-10 Criteria. J. Psychosom. Res..

[41] Martin J.L., Ancoli-Israel S. (2002). Assessment and Diagnosis of Insomnia in Nonpharmacological Intervention Studies. Sleep Med. Rev..

[42] Buysse D.J., Ancoli-Israel S., Edinger J.D., Lichstein K.L., Morin C.M. (2006). Recommendations for a Standard Research Assessment of Insomnia. Sleep.

[43] Yang M., Morin C.M., Schaefer K., Wallenstein G.V. (2009). Interpreting Score Differences in the Insomnia Severity Index: Using Health-Related Outcomes to Define the Minimally Important Difference. Curr. Med. Res. Opin..

[44] Blais F.C., Gendron L., Mimeault V., Morin C.M. (1997). Assessment of Insomnia: Validation of Three Questionnaires. Encephale.

[45] Fontes F., Goncalves M., Maia S., Pereira S., Severo M., Lunet N. (2017). Reliability and Validity of the Pittsburgh Sleep Quality Index in Breast Cancer Patients. Support. Care Cancer.

[46] Otte J.L., Rand K.L., Carpenter J.S., Russell K.M., Champion V.L. (2013). Factor Analysis of the Pittsburgh Sleep Quality Index in Breast Cancer Survivors. J. Pain Symptom Manag..

[47] Morin C.M. (2003). Measuring Outcomes in Randomized Clinical Trials of Insomnia Treatments. Sleep Med. Rev..

[48] Moul D.E., Hall M., Pilkonis P.A., Buysse D.J. (2004). Self-Report Measures of Insomnia in Adults: Rationales, Choices and Needs. Sleep Med. Rev..

[49] Savard M.H., Savard J., Simard S., Ivers H. (2005). Empirical Validation of the Insomnia Severity Index in Cancer Patients. Psychooncology.

